# Broadband reconfigurable logic gates in phonon waveguides

**DOI:** 10.1038/s41598-017-12654-3

**Published:** 2017-10-06

**Authors:** D. Hatanaka, T. Darras, I. Mahboob, K. Onomitsu, H. Yamaguchi

**Affiliations:** NTT Basic Research Laboratories, NTT Corporation, Atsugi-shi, Kanagawa 243-0198 Japan

## Abstract

The high-quality-factor mechanical resonator in electromechanical systems has facilitated dynamic control of phonons via parametric nonlinear processes and paved the development of mechanical logic-elements. However, the narrow spectral bandwidth of the resonating element constrains the available nonlinear phenomena thus limiting the functionality of the device as well as the switching speeds. Here we have developed phonon waveguides, with a two-octave-wide phonon transmission band, in which mechanical four-wave-like mixing is demonstrated that enables the frequency of phonon waves to be converted over 1 MHz. We harness this platform to execute multiple binary mechanical logic gates in parallel, via frequency division multiplexing in this broadband, where each gate can be independently reconfigured. The fidelity of the binary gates is verified via temporal measurements yielding eye diagrams which confirm the availability of high speed logic operations. The phonon waveguide architecture thus offers the broadband functionality that is essential to realising mechanical signal processors.

## Introduction

Mechanical computing was pioneered in Babbage’s difference engines in 1822^1,2^ but this platform was rendered obsolete with the emergence of the transistor in 1947^3^. Nowadays, advanced fabrication techniques have resulted in the reemergence of mechanical systems. These nano/micro-electromechanical systems (N/MEMS) consist of a tiny mechanical element, in contrast to its archaic ancestors, and the integrated electrical circuit provides the means to manipulate mechanical vibrations i.e. acoustic phonons with miniscule power consumption^4–6^. Indeed this latter prospect in particular has stimulated a flurry of activity to develop mechanical components that could be exploited in a classical nanomechanical computer^7–20^ and even in optomechanical system for quantum applications^21–24^.

A binary logic gate is at the heart of a digital computing which is constructed by wiring multiple transistors into a circuit. As a first step, the primary logic gates (AND, OR, NOT/XOR^7,10,16,17^) were replicated using N/MEMS where binary information was encoded into either the amplitude or phase of the mechanical vibration. Subsequently logic circuits were also developed either by combining mechanical elements^11^ or by spectrally multiplexing a single mechanical resonance^10^. In particular this latter approach was predicated on parametric oscillations which could be activated by nonlinearly varying a parameter of the mechanical element via electrical means^25–30^. Indeed the dynamic control of phonons in this regime provides a universal platform in which logic-circuits^10,17^, memory^8^, switches^12,17^ and a shift register^13^ can be implemented namely all the key components one would need to build a nanomechanical computer. In spite of the success of this approach it has come at the cost of a narrow operation bandwidth due to the high quality factor of the mechanical resonance which while enhancing the fidelity of the nonlinear modulation at the heart of the parametric oscillations, it limits the functionality and the processing speed of the resultant logic operations.

Here we demonstrate the above concepts using a phonon waveguide (WG) which is composed from an array of vibrating membranes that are strongly coupled and it results in their individual resonances merging to give rise to a broadband transmission band^12,31^. The broad transmission band can still host elastic nonlinearities such as third-order nonlinear effect corresponding to a cubic term of the mechanical vibration amplitude, which can be activated due to the strongly confined vibrations within the membrane array and by exciting the WG from the highly efficient piezoelectric transducers that are integrated directly into the membranes. This nonlinearity is harnessed to implement mechanical frequency mixing analogous to four-wave-mixing (FWM)^32–34^ which enables AND, OR and XOR logic gates to be executed with switching speeds up to 3 kb/s.

## Results

**Figure 1:**
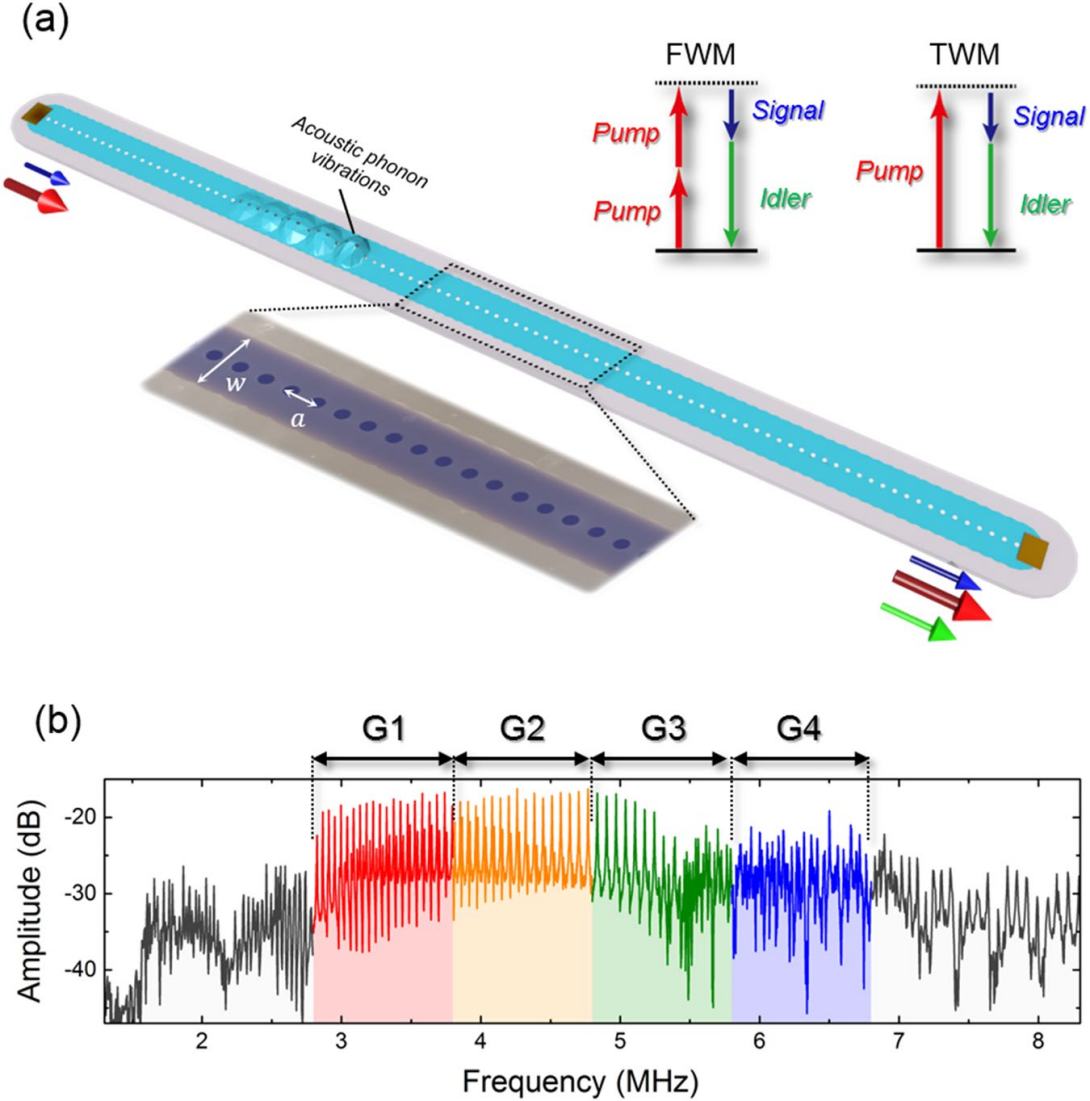
An electromechanical phonon waveguide. (**a**) A schematic of the FWM-like process in the electromechanical phonon WG. The individual membranes (light blue) in the WG are suspended above the substrate and isolated from their surroundings (grey) thus enabling acoustic phonon vibrations to be confined and guided. The left inset shows a false-colored electron microscope image of the device indicating the WG width (*w*) and the hole pitch (**a**). The right insets show energy diagrams for the FWM-like process, investigated in this report, and TWM process previously employed to execute mechanical logic circuits. In this process, a strong pump wave (red arrow) activates a third-order term in the restoring force in the phonon WG and the simultaneous excitation of a signal wave (blue arrow) stimulates the emission of a new idler wave (green arrow). (**b**) The phonon transmission spectrum in WG 1 when excited from the left edge with 1 V_rms_ and measured at the right edge via optical interferometry in a vector signal analyser. The phonon band is divided into four 1-MHz spectral regions, labelled G1, G2, G3 and G4 and highlighted in red, yellow, green and blue respectively, in order to demonstrate frequency-division-multiplexed logic gates detailed below.

The phonon WG, shown in Fig. 1(a), consists of a one-dimensional array of coupled membrane mechanical resonators made from a GaAs/AlGaAs heterostructure^12,31^. Periodically arrayed air-holes are defined on the 1-mm long WG which are used to suspend the membranes as described in Methods. The experiments were performed using two WGs having (*w*, *a*) = (34, 8) and (34, 12) in *μ*m, where *w* and *a* are waveguide width and hole pitch respectively, and hereafter they are labelled WG 1 and WG 2 respectively. The piezoelectric effect in the GaAs-based structure enables phonon vibrations to be excited by applying an alternating voltage to one of the electrodes located on both edges of the WG. The resultant phonons travel down the WG and are measured at the other edge via optical interferometry. All the experiments were performed at room temperature and in a high vacuum (2 × 10^−4^ Pa).

First the spectral response of the phonon transmission in WG 1 is investigated by exciting and measuring phonon waves at the left and right edges respectively. The phonon vibrations are observed from 1.5 to 8.2 MHz as shown in Fig. 1(b) where equidistant peaks from Fabry-Perot (FP) resonances can also be seen. Although the existence of the periodic air-holes can give rise to a phonon bandgap around 6.5 MHz in this device, the existence of another phonon branch at this frequency obscures it in the spectral domain thus permitting a continuous band of mechanical vibrations over two-octaves wide to be accessed (see Supplementary Note 1)^31^.

The WG structure can effectively confine phonon waves within the suspended membranes and in combination with the highly potent piezotransducers, it enables nonlinearities to be induced into the elastic restoring force^28,30^. The strong excitation of a *pump* phonon wave with frequency *f*_p_ activates a nonlinearity in the elastic restoring force which enables a FWM-like process to be executed. This allows an incident *signal* phonon wave with frequency *f*_s_ to interact with the pump wave which in turn generates a new *idler* phonon wave with frequency *f*_i_ = 2*f*_p_ − *f*_s_. In contrast to the parametric frequency modulation of the restoring force previously employed to implement three-wave-mixing (TWM) in a resonating electromechanical system to execute logic gates and circuits^10^, the FWM-like process is suited to the phonon WG as the frequencies of the phonon waves involved in this process are more closely spaced which enables phase matching between the different waves to be readily satisfied in addition to energy conservation.

It should be noted that idler phonons in principle can also be generated via TWM process. In practice this process can be weaker than the FWM-like process in mechanical systems. This is because at equilibrium the mechanical membranes are centre symmetric, namely their underlying potential is symmetric at zero displacement, and in this limit the TWM process is unavailable^35^. Further, the idler generated from TWM, for the signal and pump frequencies used in this study, i.e. *f*_i_ = *f*_p_ − *f*_s_ would appear below the cut-off frequency (1.5 MHz) of the phonon WGs thus making these idlers, even if TWM was possible for this system, inaccessible.

**Figure 2:**
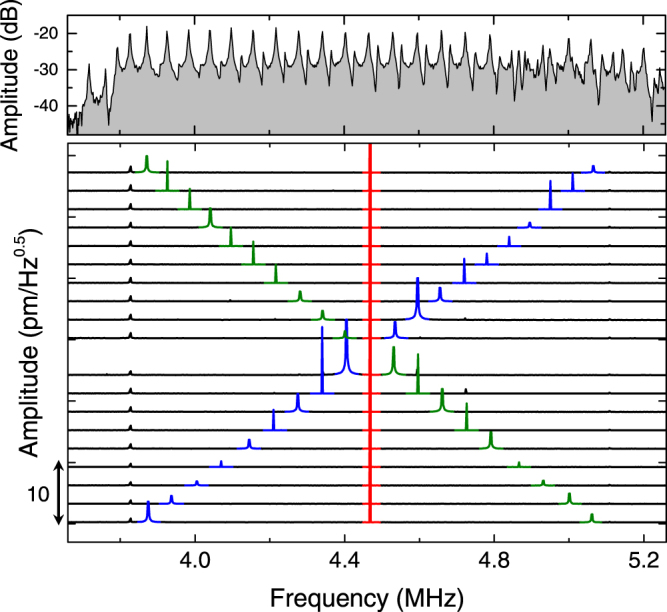
Frequency conversion of phonon waves. The spectral response of phonon WG 2 when excited with a fixed pump excitation at 4.468 MHz and an amplitude of 1 V_rms_ (red) whilst simultaneously the signal excitation is swept from 3.8 to 5.1 MHz with an amplitude of 0.35 V_rms_ (blue). Note that the generated idler (green) shifts from high to low frequencies as the signal is swept from low to high frequencies in order to conserve energy. The upper panel shows the frequency response of WG 2 when excited from the left edge with a 1 V_rms_ amplitude and measured at the right edge.

In order to confirm the availability of FWM-like process in the phonon WG architecture, the spectral response of WG 2 is monitored by simultaneously activating a weak signal (blue) and a strong pump wave (red). The results of this measurement shown in the lower panel of Fig. 2 reveal the generation of an idler wave (green) from the frequency interaction between the signal and pump waves. Moreover the frequency of the idler wave can be continuously tuned over 1 MHz as the signal excitation is adjusted indicating the availability of broadband frequency conversion in contrast to the narrow band frequency conversion via TWM previously demonstrated in a resonating mechanical system^10^ (see Supplementary Note 2 and Supplementary Fig. 2). Momentum conservation is crucial for efficient frequency mixing, in addition to energy conservation requirements, and to achieve this in the phonon WG requires a linear dispersion relation^32^. For WG 2 an almost linear dispersion relation between 4.3–4.6 MHz exists in the phonon transmission band where the equally spaced FP resonances are observed as shown in the upper panel of Fig. 2. In this spectral window, the idler amplitude is enhanced when the signal, pump and idler waves overlap with the FP resonances and it corresponds to phase matching^34,36,37^ as detailed in Supplementary Note 3 and Supplementary Fig. 3.

The FWM-like process can be harnessed to implement the AND, OR and XOR fundamental logic gates. In this approach two signal waves at *f*_s1_ and *f*_s2_ = *f*_s1_ + Δ are used to encode binary logical inputs and the generated idler wave yields the binary output with the presence (absence) of a phonon vibration being defined as 1 (0). By adjusting the number, frequency and phase of the pump waves used to activate the FWM-like process, a range of idler waves can be generated whose signal wave dependence enables AND, OR and XOR gates to be created in the phonon WG. In practice the primary logic gates are implemented in one of four spectral regions defined between 2.8–6.8 MHz in WG 1, henceforth labelled G1, G2, G3, G4 and detailed in Fig. 1(b), yielding a Boolean logic gate array in frequency space. In this report, the phonon band beyond G4 is not employed as the vibration amplitudes are small in this region due to the poor excitation of the piezoelectric transducer. However this high-frequency region can be accessed, for logic operations, by optimizing the transducer geometry so that is more effective at activating the corresponding shorter wavelength phonons.

**Figure 3:**
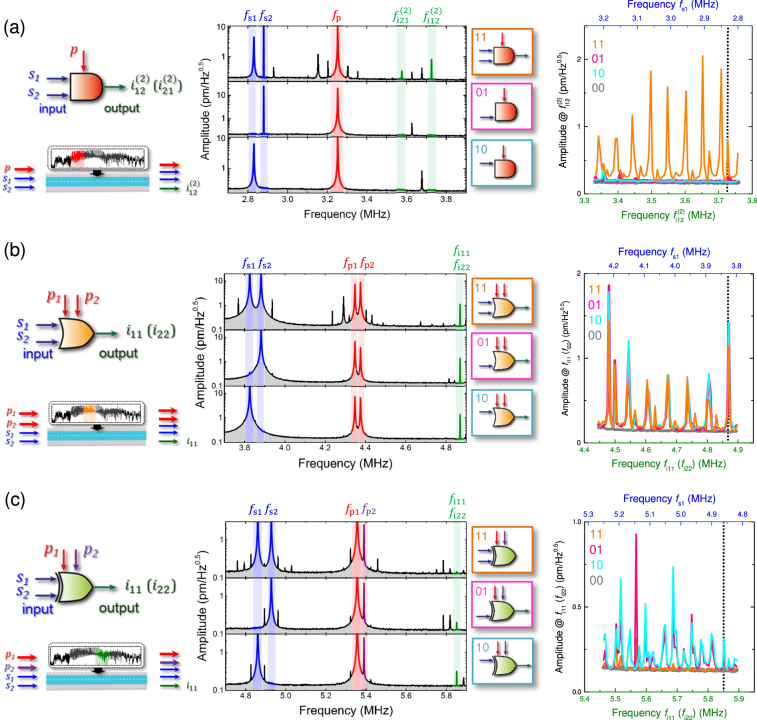
Frequency-division-multiplexed logic gates in the phonon WG. The output spectra of (**a**) AND, (**b**) OR and (**c**) XOR logic gates executed in spectral windows G1, G2 and G3 in WG 1 respectively as detailed in Fig. 1(b). Left: The input/output configurations of the logic gate being executed via the phonon excitations in the WG where the signal, pump and idler are coloured blue, red (or purple to imply a phase shift) and green respectively throughout. Middle: All the logic gates are executed as a function of signal input configurations in the WG where signal 1 (*f*_s1_) and signal 2 (*f*_s2_) being on and off respectively yields the binary input 10, *f*_s1_ = off *f*_s2_ = on yields 01, both signals being on gives 11 and the corresponding input/output configurations of the logic gates are shown in the right insets. Note that numerous other peaks are generated in the WG when all the input excitations are activated which corresponds to different mixing combinations between the signal, pump and idler waves. Right: The output logical idlers can be generated over a broad range of frequencies as the signal frequencies are swept for the different input configurations of 00 (grey), 10 (light blue), 01 (pink) and 11 (orange). The dotted line indicates the idlers described in the middle panel.

In order to implement an AND gate, a pump wave (*f*_p_) and two signal waves (*f*_s1_ and *f*_s2_) are simultaneously injected into the WG resulting in two idler waves being generated at ${f}_{{\rm{i}}1}^{\mathrm{(1)}}=2{f}_{{\rm{p}}}-{f}_{{\rm{s}}1}$ and ${f}_{{\rm{i}}2}^{\mathrm{(1)}}=2{f}_{{\rm{p}}}-{f}_{{\rm{s}}2}$ from the frequency mixing. These idler waves then serve as seeds for further FWM-like process yielding secondary idler waves at ${f}_{{\rm{i}}12}^{\mathrm{(2)}}=2{f}_{{\rm{i}}1}^{\mathrm{(1)}}-{f}_{{\rm{i}}2}^{\mathrm{(1)}}$ and ${f}_{{\rm{i}}21}^{\mathrm{(2)}}=2{f}_{{\rm{i}}2}^{\mathrm{(1)}}-{f}_{{\rm{i}}1}^{\mathrm{(1)}}$. The secondary idlers can only be observed when both signal waves are injected into the WG thus naturally leading to an AND gate (see Supplementary Note 4.1). In the middle panel of Fig. 3(a), this gate is experimentally implemented in region G1 with signal waves *f*_s1_ = 2.830 MHz and *f*_s2_ = 2.880 MHz namely Δ = 50 kHz (blue), pump wave *f*_p_ = 3.254 MHz (red) and the secondary idler waves (green) are observed at ${f}_{{\rm{i}}12}^{\mathrm{(2)}}$ = 3.728 MHz and ${f}_{{\rm{i}}21}^{\mathrm{(2)}}$ = 3.578 MHz only when both signals are activated. On the other hand, when either of the two signals is not excited, no secondary idler waves are generated. Thus the secondary idler response corresponds to an AND gate which can be executed between 3.35–3.75 MHz as shown in the right panel of Fig. 3(a) (see Supplementary Fig. 4 for more details).

Next to implement an OR gate, spectrally degenerate idler waves are generated from two pump waves at *f*_p1_ and *f*_p2_ = *f*_p1_ + Δ/2 in addition to the two signal waves (*f*_s1_ and *f*_s2_). This results in four idler waves of which two can be made degenerate at frequency *f*_i11_ = 2*f*_p1_ − *f*_s1_ = 2*f*_p2_ − *f*_s2_ = *f*_i22_ by appropriately tuning *f*_p2_ (see Supplementary Note 4.2). Consequently this enables the degenerate idlers to be observed when either or both signals are injected into the WG yielding an OR gate. Experimentally two signals *f*_s1_ = 3.825 MHz and *f*_s2_ = 3.881 MHz (blue) and two pumps *f*_p1_ = 4.347 MHz and *f*_p2_ = 4.375 MHz namely Δ = 56 kHz (red) are injected into G2 which results in output idler waves at *f*_i11_ = *f*_i22_ = 4.869 MHz (green) as shown in the middle panel of Fig. 3(b). This measurement reveals the OR gate can be successfully executed between 4.5–4.9 MHz as shown in the right panel of Fig. 3(b), when the signal frequencies are swept, from the degenerate idlers generated by the precisely tuned dual pump excitations (see Supplementary Fig. 5 for more details).

Finally to realise the XOR gate, destructive interference between the degenerate idler waves, used for the OR gate, is utilised. Specifically two pump waves tuned to yield degenerate idlers, but now with a *π*/2 phase difference, are applied to the WG which causes the idler waves to interfere and cancel out resulting in the XOR gate operation (see Supplementary Note 4.3 and Supplementary Fig. 6). In the experiment, two pumps *f*_p1_ = 5.356 MHz and *f*_p2_ = 5.390 MHz (i.e. Δ = 67.5 kHz) with a relative *π*/2 phase shift (red and purple in the middle panel of Fig. 3(c)) and two signals *f*_s1_ = 4.860 MHz and *f*_s2_ = 4.928 MHz are injected into region G3 of WG 1. This results in the degenerate idler wave at *f*_i11_ = *f*_i22_ = 5.852 MHz being eliminated, as shown in the middle panel of Fig. 3(c), whereas excitation of either one of the two signals yields an idler wave output. The XOR output can also be observed between 5.45–5.9 MHz in the phonon WG when the signal wave frequencies are swept as shown in the right panel of Fig. 3(c) (see Supplementary Fig. 7 for more details).

The AND, OR and XOR gates can be executed in all four spectral regions G1-G4 in the phonon WG by simply adjusting the pump conditions. More importantly, the idler outputs can be used as signal inputs for further logic operations that would enable the realisation of a series of logic gates and construction of complex logic circuits. Consequently multiple and reconfigurable logic gates via a mechanical FWM-like process can be implemented over the broad transmission band in the phonon WG.

**Figure 4:**
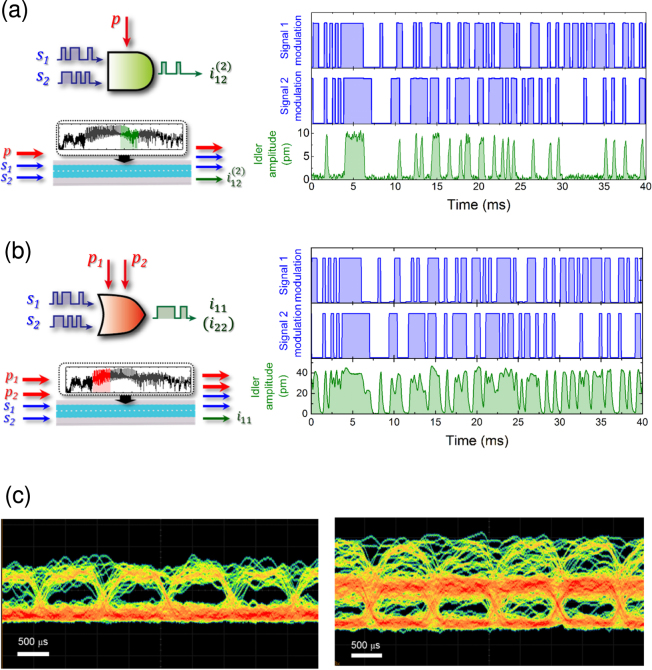
High speed operation of mechanical logic gates. (**a**) and (**b**) The temporal response of AND and OR gates, schematically depicted in the left panels, in regions G3 and G1 of WG 1 respectively. The two input signals waves are modulated by a PRBS at 3 kb/s, shown in the right panels, along with the idler encoding the logical output. (**c**) The eye diagram for the AND and OR gates operated in region G2 when the input signals are modulated by a PRBS at 1 kb/s. The eye openings indicate the successful implementation of mechanical logic gates at high speed in the phonon WG.

The broadband phonon WG also offers the prospect of high speed logic gates. To that end the AND gate was realised in region G3 in WG 1 with two signal waves at *f*_s1_ = 5.010 MHz and *f*_s2_ = 5.077 MHz which were rapidly amplitude modulated by a pseudo-random bit sequence (PRBS) at 3 kb/s and a continuous wave (CW) pump at *f*_p_ = 5.322 MHz that were simultaneously injected into the WG. The output idler at ${f}_{{\rm{i}}12}^{\mathrm{(2)}}$ = 5.702 MHz is only generated during the presence of both signal inputs, as shown in the right panel of Fig. 4(a), and thus it can successfully encode the AND gate at 3 kb/s. Additionally the OR gate was realised in region G1 in WG 1 with two signal waves injected at *f*_s1_ = 3.010 MHz and *f*_s2_ = 3.060 MHz (i.e. Δ = 50 kHz) which again were rapidly amplitude modulated by the PRBS at 3 kb/s and two CW pumps injected at *f*_p1_ = 3.279 MHz and *f*_p2_ = 3.304 MHz. Now the output idler at *f*_i11_ = *f*_i22_ = 3.548 MHz is only eliminated if both signal inputs are absent as shown in the right panel of Fig. 4(b) and thus it can also encode the OR gate at high speed. It should be noted that the OR idler’s amplitude varies when only *f*_s2_ is active in contrast to when *f*_s1_ or both *f*_s1_ and *f*_s2_ are activated and is due to idler conversion efficiencies being different for the two pumps. Note that the idler amplitudes differ between the AND and OR gates since they originate from different spectral regions of the phonon transmission band where the shorter wavelengths in region G3 exhibits smaller vibrations than in G1 as detailed in Fig. 1(b).

The fidelity of the high speed AND and OR logic gates can be evaluated via the eye pattern acquired from the above measurement protocol with multiple runs of the PRBS at 1 kb/s and is shown in Fig. 4(c) in the left and right panels respectively. The eye openings indicate that the AND gates can be successfully executed even with the presence of multiple input phonon waves that yield a rich idler spectrum. Note that the eye opening is less clearly defined for the OR gate as consequence of the differing idler conversion efficiencies between the two pumps as detailed above.

**Figure 5:**
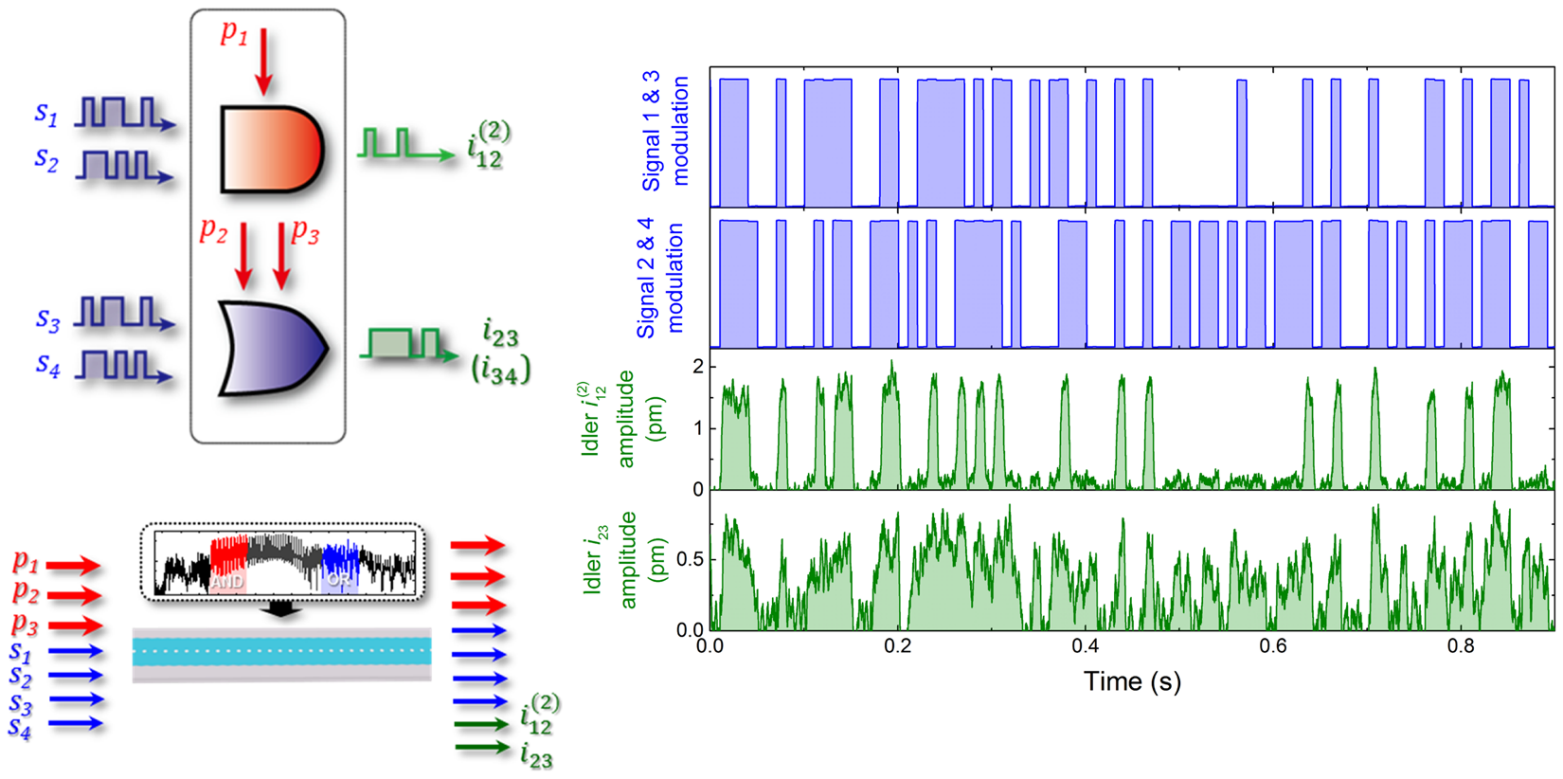
Parallel logic gates in the phonon WG. The temporal response of both AND and OR gates, depicted schematically in the left panels, when implemented simultaneously in WG 1. The AND gate is executed in G1 with two randomly amplitude modulated input signals *f*_s1_ = 2.940 MHz and *f*_s2_ = 2.990 MHz and one CW pump *f*_p1_ = 3.254 MHz yielding the AND idler ${f}_{{\rm{i}}12}^{(\mathrm{2)}}$ = 3.618 MHz. The OR gate is executed in G4 with the two input signals *f*_s3_ = 6.020 MHz and *f*_s4_ = 6.110 MHz that have the same random amplitude modulation as the AND gate and two CW pumps *f*_p2_ = 6.315 MHz and *f*_p3_ = 6.360 MHz which yield an OR idler at *f*_i23_ = *f*_i34_ = 6.610 MHz. The right panels show the temporal response of the randomly amplitude modulated input signals and the resultant output AND, OR logical idlers.

Another consequence of the broad transmission band in the WG is the possibility to execute logic gates in parallel in the different gate regions G1-G4 as detailed in Fig. 1(b) by frequency-division multiplexing the input signal and pump waves. This concept is demonstrated in WG 1 with the AND and OR gates being implemented simultaneously. Specifically two signals waves *f*_s1_ = 2.940 MHz and *f*_s2_ = 2.990 MHz, which are randomly amplitude modulated, and one CW pump *f*_p1_ = 3.254 MHz are injected in the WG to generate the output from a secondary idler at ${f}_{{\rm{i}}12}^{(\mathrm{2)}}$ = 3.618 MHz for the AND gate in G1 (see the left insets of Fig. 5). Simultaneously two additional signal waves *f*_s3_ = 6.020 MHz and *f*_s4_ = 6.110 MHz with the same random amplitude modulation and two CW pumps *f*_p2_ = 6.315 MHz and *f*_p3_ = 6.360 MHz are injected into the WG to generate the idler at *f*_i23_ = *f*_i34_ = 6.610 MHz for the OR gate in G4 (see the left insets of Fig. 5). The temporal response of the amplitudes of the output idlers ${f}_{{\rm{i}}12}^{(\mathrm{2)}}$ and *f*_i23_ (or *f*_i34_), acquired concurrently and shown in the right panels of Fig. 5, confirms that they can successfully encode AND and OR gates simultaneously. It should be noted that smaller amplitudes are observed when the AND and OR gates are executed simultaneously in contrast to when they are individually operated. For the parallel operation a total of seven input excitations were applied to the phonon WG. The large voltage associated with the sum of these excitations can trigger parasitic nonlinear effects for instance nonlinear damping^38^, nonlinear mode coupling^39^, and even current leakage across the piezoelectric transducer thus limiting its efficiency where all these effects can lead to a reduction in the output idler amplitudes.

Consequently this result indicates that frequency-division-multiplexing can be applied to the signal and pump waves injected into the phonon WG to realise a spectral logic array in which Boolean logic gates can be executed in parallel. The parallel operation scheme employed here yields one logic gate from 5 free-spectral-ranges (FSRs) of the FP resonance peaks with 2 FSRs for 2 signals, at least 1 FSR for the pump and finally 2 FSRs for the 2 idlers. The continuous band in WG 1 spans 6.7 MHz (1.5 MHz–8.2 MHz) which approximately contains 80 FSRs, and for the minimal AND case it can permit 16 logic gates to be implemented in parallel.

## Discussion

The key merit of the phonon WG is its broad transmission band which allows the operation speed of mechanical logic gates to be increased up to ~3 kb/s. In spite of this advance, and from the view point of practical applications, this speedup is still insufficient and several orders of magnitude of further enhancement is needed.

The current device is confined to the 1.5–8.2 MHz operation frequency of the transmission band and the switching speed is limited to 4 kHz i.e. half the FP resonance bandwidth. Both of these properties need to be improved before the phonon WG architecture can be considered as a practical system for information signal processing. First to increase the frequency of the transmission band requires further miniaturisation of the WG down to submicron scales. In fact the operation frequency is dependent on the inverse square of the WG width *w* and reducing this to 2 *μ*m would increase its frequency to the GHz order which in principle could yield switching speeds of ~0.1 Gb/s. However the key to this is a flat transmission band without FP resonances which will enable the full bandwidth to be harnessed to increase the switching speeds which could be achieved with long WGs without the reflecting edges. The low conversion efficiencies likely to emerge due to the absence of FP resonance will then need to be compensated for by careful engineering of the phonon dispersion relations that would permit the phase matching needed for the frequency mixing process.

Finally the power consumption for a bit processing is of fundamental importance to evaluate the performance of the phonon WG. The energy consumed by the underlying mechanical vibration can be estimated from 4*π* ^3^ *mf* ^3^ *z* ^2^/*Q*, where *m* is effective mass of the device (~10 ng), *f* is operation frequency (~5 MHz), *z* is vibration amplitude (~50 pm) and *Q* is quality factor (~500) which approximately yields 7 pW thus 2 fJ per switch at 3 kb/s thus providing a meaningful alternative to the CMOS logic cell (0.1 fJ)^11^. This mechanical power consumption can be further reduced by employing two-dimensional materials such as graphene^39^ and monolayer transition metal dichalcogenides^40,41^ due to their ultralow mass where the latter are even piezoelectrically active^41^. Consequently there is further scope to optimizing the phonon WG which could lead to even lower power consumption for logic operations in this architecture.

A phonon WG is developed in which frequency-division-multiplexed logic gates via mechanical FWM-like process are demonstrated which are not only reconfigurable on the fly they can also be operated at kHz speeds. The prospect of exploiting these techniques paves the way towards the realization of novel nanomechanical computers with low power consumption and in highly-functional optomechanical systems.

## Methods

The phonon WGs were constructed from a GaAs/AlGaAs heterostructure by isotropically and selectively etching a 3 *μ*m thick Al_0.65_Ga_0.35_As sacrificial layer through the periodic air-holes that were defined through a 5 nm GaAs layer on top of 95 nm Al_0.27_Ga_0.73_As and 100 nm n-GaAs layers with HF(5%):H_2_O(95%) solution. The 40 min selective etching time also determined the WG’s width to be 34 *μ*m. The 80 nm thick Au gates were located on both edges of the WGs and they formed the top electrode of the piezoelectric stack that was used to activate the phonon waves in the WG.

The application of alternating electric voltage from a signal generator (NF Wavefactory 1974) to one of the electrodes induces bending mechanical vibrations in the WG due to the piezoelectric effect. The resultant vibration travels down the WG and is optically detected in a He-Ne laser Doppler interferometer (Neoark MLD-230V-200). The spectral responses in Figs 1(b), 2 and 3(a–c) are obtained by demodulating the output from the interferometer in a vector signal analyzer (HP 89410A) or a lock-in amplifier (Zurich Instruments UHFLI). The temporal responses in Figs 4 and 5 are obtained by amplitude modulating the signal generator with 2^14^-1 PRBS non-return-to-zero from a pulse pattern generator (Keysight Technologies 81110A) and demodulating the interferometer in a lock-in amplifier followed by an oscilloscope (Keysight Technologies DSO9104H).

## Electronic supplementary material

Supplementary information
