# Supplementary material for: Broadband reconfigurable logic gates in phonon waveguides
Source: Sci Rep. 2017 Oct 6;7:12745. doi: 10.1038/s41598-017-12654-3 (PMC5630582; doi:10.1038/s41598-017-12654-3)
Supplement: Supplementary file 1 — Supplementary information [file 41598_2017_12654_MOESM1_ESM.pdf]

Supplementary Information

Broadband reconfigurable logic gates in phonon waveguides

D. Hatanaka, T. Darras, I. Mahboob, K. Onomitsu and H. Yamaguchi

NTT Basic Research Laboratories, NTT Corporation, Atsugi-shi, Kanagawa 243-0198, Japan

Supplementary Note 1 Dispersion relation in WG 1

The dispersion relation in WG 1 can be extracted from a finite-element-method simulation as shown in Supplementary Fig. 1. This reveals the first phonon band (red) starting at 1.6 MHz approaches the first Brillouin zone (BZ) edge at 6.5 MHz where a bandgap exists to the third phonon band (blue) which spans 5.7-8.4 MHz and overlaps the bandgap away from the first BZ edge. Consequently the mechanical vibrations supported by this band can mask the bandgap as experimentally observed in the transmission spectrum in Fig. 1(b).

Supplementary Note 2 Broadband frequency conversion

In order to demonstrate the availability of broadband frequency conversion via frequency mixing such as four-wave-mixing (FWM) in the phonon waveguide (WG), the pump wave is fixed on the Fabry-Perot (FP) resonance peak at 4.468 MHz and the signal wave is swept in the transmission band of WG 2 between 3.8-5.1 MHz. The resultant idler phonon wave generated in the transmission band is shown in Supplementary Fig. 2. As the signal frequency passes through the FP resonances, the amplitude of the idler wave is enhanced, due to the field enhancement, and it reproduces the FP resonance spectral structure observed in the transmission measurements.

Supplementary Note 3 Idler amplification via Fabry-Perot resonance

The FP resonances in the WG can be used to enhance the efficiency of the FWM-like process. Supplementary Fig. 3(a) shows the idler amplitude as a function of pump excitation voltage. The idler (green lines in Supplementary Fig. 3(b)) generation efficiency is strongly dependent on the frequency mismatch of it and the signal and pump waves (blue and red lines in Supplementary Fig. 3(b)) with respect to the FP resonances. For instance when both the signal and pump excitation frequencies are detuned from the FP frequencies (grey points in Supplementary Fig. 3(a) and bottom panel in Supplementary Fig. 3(b)), the idler amplitude is markedly less than when either the signal or pump waves are tuned onto the FP resonances (green and blue points in Supplementary Fig. 3(a) and the middle panels in Supplementary Fig. 3(b) respectively). Finally if both the signal and pump excitations are tuned onto the FP resonances, the idler amplitude exhibits a further amplification of 7 dB as detailed by the red points in Supplementary Fig. 3(a) and the top panel in Supplementary Fig. 3(b). This enhancement in the idler generation is due to the reduced phase mismatch between the signal, pump and idler waves, in addition to energy conservation, which is almost entirely eliminated when all the waves are located at the spectral positions of the FP resonances.

Supplementary Note 4 Frequency dependence of the primary logic gates

The broadband response of the idler generation, detailed in Supplementary Note 2, indicates that the primary logic gates executed in the main text are not limited to narrow spectral regions as is the case for mechanical resonator based logic gates. The spectral dependence of the logic gates can be predicted from the details of the FWM-like process used to generate them and is described below.

Supplementary Note 4.1 AND gate

As detailed in the main text, the AND gate is executed by employing two signal waves into which binary information is encoded and a single pump wave (f_p) which generate the second-order idler waves. As a first step to this gate, signal wave 1 (f_s1) is activated and signal wave 2 (f_s2) is inactive corresponding to the binary input 10. In this configuration an idler from f_s1 is generated when the pump is activated as $f_\mathrm{i1}^{(1)}$ = $2f_\mathrm{p}$ - f_s1, namely the idler has a negative variation with respect to the signal wave as depicted in the left panel of Supplementary Fig. 4(a) via the blue, red and green lines (throughout) for the signal, pump and idler waves respectively, and experimentally confirmed in the left panel of Supplementary Fig. 4(b). A similar observation is also made in the opposite configuration, namely the logical input 01 yielding the idler $f_\mathrm{i2}^{(1)}$ = $2f_\mathrm{p}$ - f_s2 as depicted in the middle panel of Supplementary Fig. 4(a) and experimentally confirmed in the middle panel of Supplementary Fig. 4(b). Note that the $f_\mathrm{i1}^{(1)}$ and $f_\mathrm{i2}^{(1)}$ idlers do not overlap. Finally with both signal waves activated corresponding to the logical input 11, second-order idlers ($f_\mathrm{i12}^{(2)}$ and $f_\mathrm{i21}^{(2)}$) are generated as:

$$\begin{aligned} f_\mathrm{i12}^{(2)} &= 2f_\mathrm{i1}^{(1)} - f_\mathrm{i2}^{(1)} \\ &= 2(2f_\mathrm{p} - f_\mathrm{s1}) - (2f_\mathrm{p} - f_\mathrm{s2}) \\ &= 2f_\mathrm{p} - 2f_\mathrm{s1} + f_\mathrm{s2}, \end{aligned} \tag{S1}$$

and,

$$\begin{aligned} f_\mathrm{i21}^{(2)} &= 2f_\mathrm{i2}^{(1)} - f_\mathrm{i1}^{(1)}, \\ &= 2(2f_\mathrm{p} - f_\mathrm{s2}) - (2f_\mathrm{p} - f_\mathrm{s1}) \\ &= 2f_\mathrm{p} - 2f_\mathrm{s2} + f_\mathrm{s1}, \end{aligned} \tag{S2}$$

and are depicted by the dark green lines in the right panel of Supplementary Fig. 4(a) and are experimentally confirmed in the right panel of Supplementary Fig. 4(b). Since these second-order idlers only emerge when both signal waves are activated they naturally enable the realisation of the AND gate. Importantly since the second-order idlers wave can be tuned across the entire transmission band in the WG, by adjusting the signal frequency, they enable this gate to be executed over a wide range of frequencies.

Supplementary Note 4.2 OR gate

Again as detailed in the main text, the OR gate is realised by employing two signal waves with f_s2 = f_s1 + Δ into which binary information is encoded and two pump waves with f_p2 = f_p1 + $\Delta/2$ which generate the OR idlers. Specifically activating f_s1 corresponding to the logical input 10 yields two idlers with

$$\begin{aligned} f_\mathrm{i11} &= 2f_\mathrm{p1} - f_\mathrm{s1}, \\ f_\mathrm{i21} &= 2f_\mathrm{p2} - f_\mathrm{s1}, \end{aligned} \tag{S3}$$

which are depicted in the left panel of Supplementary Fig. 5(a) and experimentally confirmed in the left panel of Supplementary Fig. 5(b). Conversely activating f_s2 corresponding to the logical input 01 yields the idlers

$$\begin{aligned} f_\mathrm{i12} &= 2f_\mathrm{p1} - f_\mathrm{s2}, \\ f_\mathrm{i22} &= 2f_\mathrm{p2} - f_\mathrm{s2}, \end{aligned} \tag{S4}$$

which are depicted in the middle of Supplementary Fig. 5(a) and experimentally confirmed in the middle of Supplementary Fig. 5(b). Crucially it can now be shown using equations (S3) and (S4)

$$\begin{aligned} f_\mathrm{i22} &= 2f_\mathrm{p2} - f_\mathrm{s2} \\ &= 2(f_\mathrm{p1} + \Delta/2) - (f_\mathrm{s1} + \Delta) \\ &= 2f_\mathrm{p1} - f_\mathrm{s1} \\ &= f_\mathrm{i11}, \end{aligned} \tag{S5}$$

namely these idlers are degenerate. Therefore activating both signal waves corresponding to the logical input 11 yields the idler response depicted in the right panel of Supplementary Fig. 5(a) with the degenerate idlers given by the dark green line which is experimentally confirmed in the right panel of Supplementary Fig. 5(b). Naturally these idlers are always present if either or both signal waves are activated and thus they naturally lead to an OR gate which can be executed over a broad range of frequencies in the phonon WG.

Supplementary Note 4.3 XOR gate

As detailed in the main text, the XOR gate is executed in a similar configuration to the OR gate except the pump phase is adjusted so that the degenerate idlers destructively interfere as shown explicitly in Supplementary Fig. 6. Indeed when the phase difference between the two pumps is $\pi/2$ or $3\pi/2$, the degenerate idlers are eliminated from the destructive interference.

By fixing the phase difference between the two pumps at $\pi/2$ and activating f_{s1} (f_{s2}) corresponding to the logical 10 (01) yields the idler response shown in the left (middle) panel of Supplementary Fig. 7(a) which is extracted from equations (S3), (S4) and (S5) with the degenerate idler captured by the dark green line and the pump with the phase difference in the purple line. The corresponding experimental response is shown in the left (middle) panel of Supplementary Fig. 7(b). Finally activating both signal waves corresponding to the logical input 11 yields the idler response depicted in the right panel of Supplementary Fig. 7(a) with the degenerate idler being eliminated which is experimentally confirmed in the right panel of Supplementary Fig. 7(b). Naturally this idler encapsulates the XOR gate as it is absent when both signal waves are activate and it can be executed over a broad range of frequencies in the phonon WG.

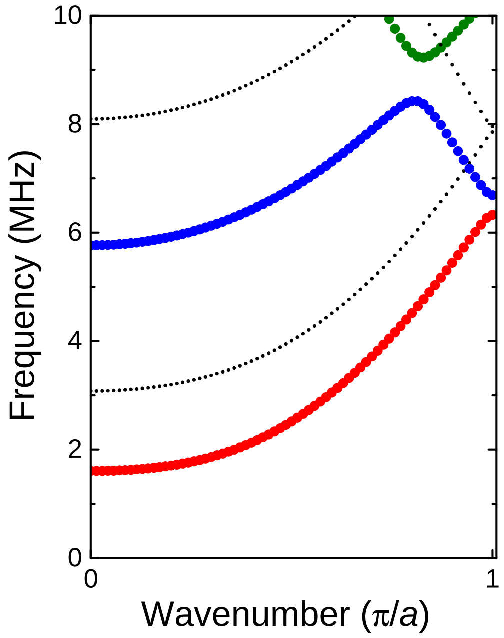

SUPPLEMENTARY FIGURE 1: **Dispersion relation in WG 1.** The first, third and fifth phonon bands are shown in red, blue and green dotted lines respectively. Black dotted lines indicate the second and fourth bands which are not excited due to the mismatch between the location of the node of these modal shapes and the piezotransducer.

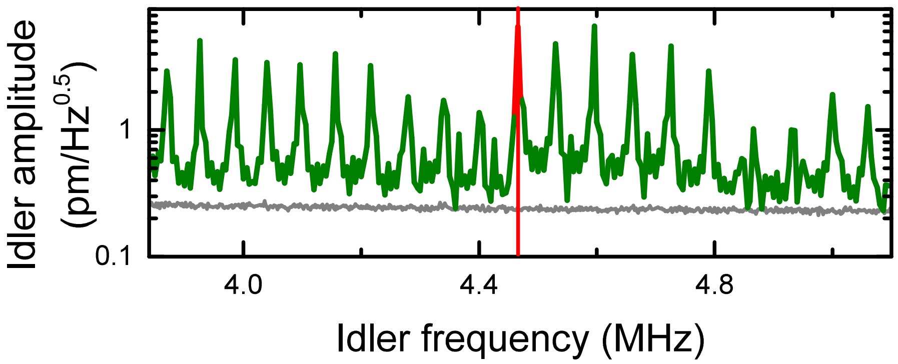

SUPPLEMENTARY FIGURE 2: **Broadband idler generation.** The spectral dependence of the idler waves (green) generated by sweeping the signal wave from 3.8 to 5.1 MHz with an amplitude of 0.35 V_{rms} whilst the pump wave is excited at a fixed frequency of 4.468 MHz (red line) and an amplitude of 1.0 V_{rms} in WG 1. The grey line shows the background noise in the WG measured via optical interferometry and demodulated in a spectrum analyser in the absence of FWM-like process.

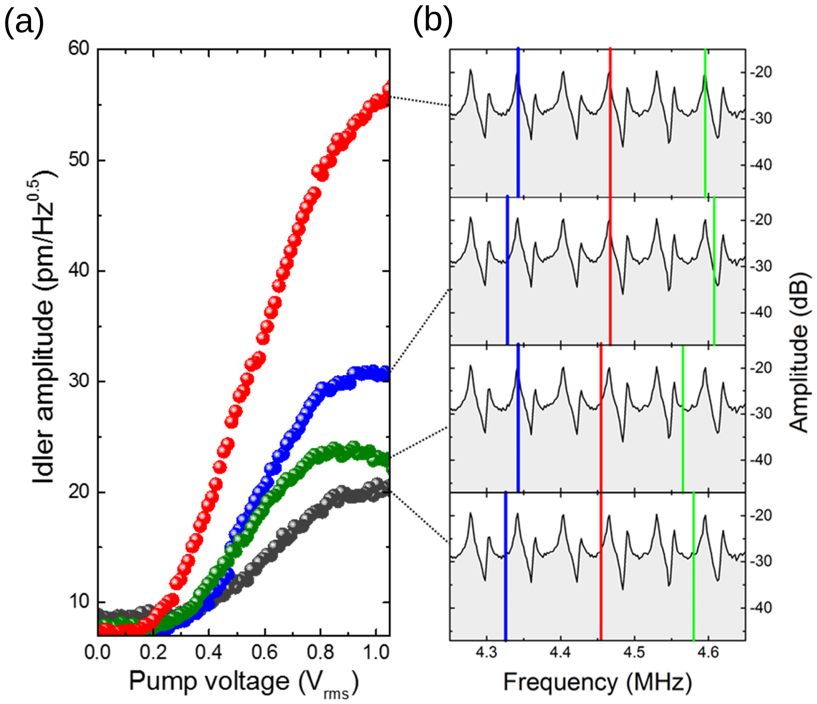

SUPPLEMENTARY FIGURE 3: **The pump voltage dependence of the idler amplitude.** (**a**) The pump amplitude dependence of the idler amplitude for various combinations of signal and pump excitation frequencies that are detailed in (**b**). (**b**) The peak structure corresponds to the FP resonances in the WG, acquired from the transmission measurement in the upper panel of Fig. 2, and all measurements were conducted with a signal amplitude of 0.35 V_{rms}.

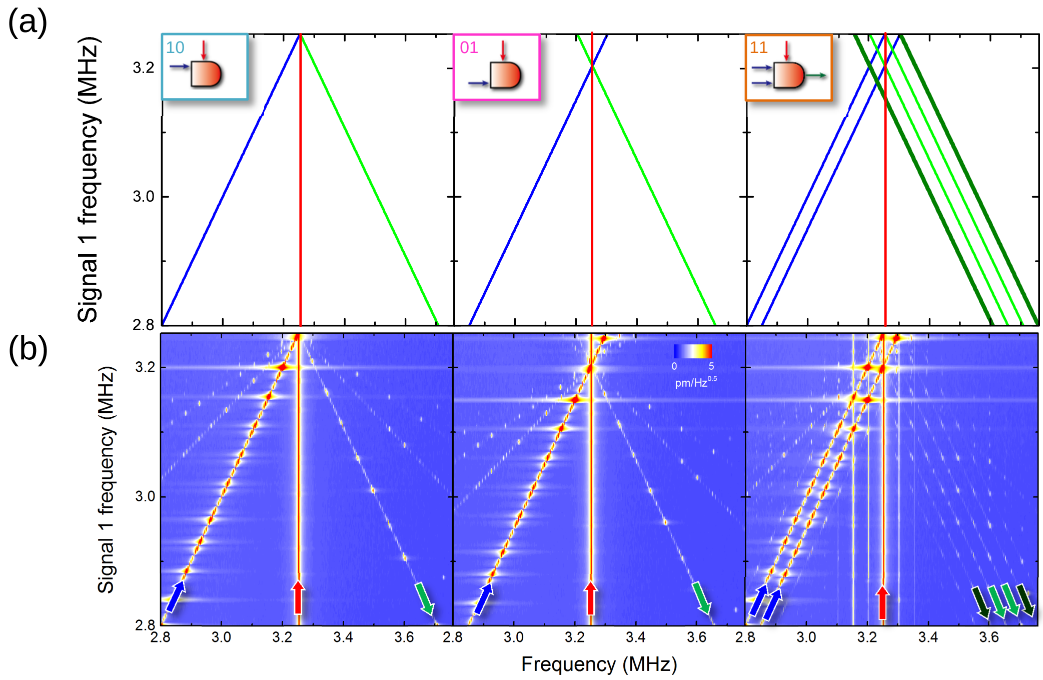

SUPPLEMENTARY FIGURE 4: **AND gate.** (**a**) The spectral dependence of the idler waves (green and dark green lines), as detailed above, generated from a single pump wave (red line) at fixed frequency as function of two signal waves (blue lines) when either is on/off (left panel) or off/on (middle panel) or when both are on (right panel) corresponding to logical inputs 10, 01 and 11 respectively. In the latter configuration, second-order idlers (dark green lines) are also generated as detailed in equations (S1) and (S2) which are used to execute the AND gate. (**b**) The experiments were conducted in region G1 of WG 1 with the 1.5 V_{rms} pump wave fixed at 3.254 MHz (red arrows). The signals waves (blue arrows) are injected with an amplitude of 1.5 V_{rms} with $f_{s2} = f_{s1} + 50$ kHz in the frequency range of 2.80-3.25 MHz. The second-order idler waves (dark green arrows) are generated between 3.25-3.75 MHz only in the presence of both signals (right panel), enabling the AND gate to be realised in the phonon WG. The insets show the signal and pump configurations of the logic gate being performed.

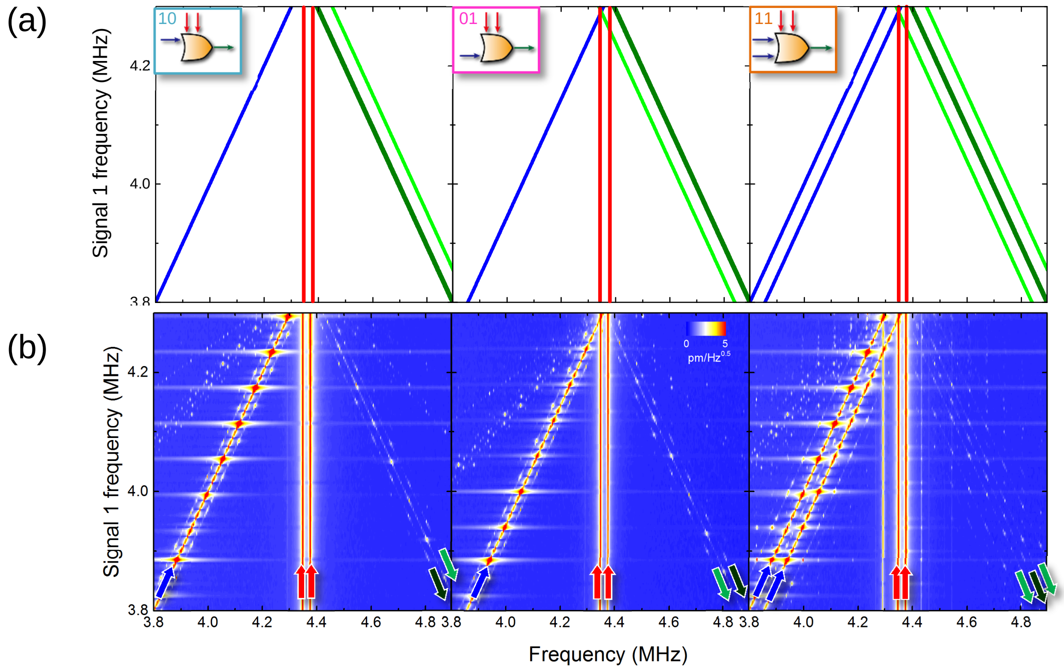

SUPPLEMENTARY FIGURE 5: **OR gate.** (**a**) The spectral dependence of the idler waves (green and dark green lines), as detailed above, generated from two pump waves (red lines) at fixed frequencies as function of two signal waves (blue lines) when either is on/off (left panel) or off/on (middle panel) or when both are on (right panel) corresponding to logical inputs 10, 01 and 11 respectively. In the latter configuration, degenerate idlers (dark green line) are present as detailed in equations (S5) which are used to execute the OR gate. (**b**) The experiments were conducted in region G2 of WG 1 with two 1 V_{rms} pump waves fixed at f_{p1} = 4.347 MHz and f_{p2} = 4.375 MHz i.e. $\Delta/2$ = 28 kHz (red arrows). Two signals waves (blue arrows) are injected with an amplitude of 1.5 V_{rms} at $f_{s2} = f_{s1}$ + 56 kHz and in the frequency range of 3.8-4.3 MHz (blue arrows). The degenerate idler waves $f_{i11} = f_{i22}$ (dark green line) are always observed between 4.5-4.9 MHz when either or both signal waves are activate, yielding the OR gate in the phonon WG. The insets show the signal and pump configurations for the logic gate being performed.

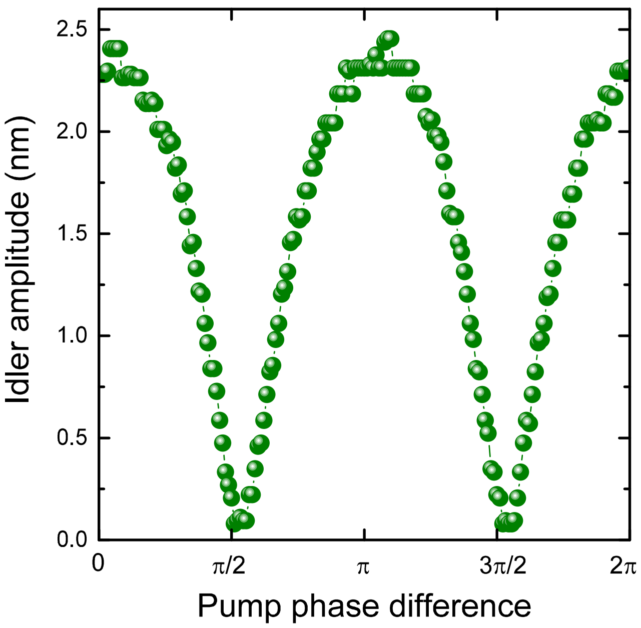

SUPPLEMENTARY FIGURE 6: **The pump phase dependence of the spectrally degenerate idler wave.** The degenerate idler amplitude at $f_{i11} = f_{i22}$= 5.687 MHz when the phase difference between f_{p1} = 5.356 MHz and f_{p2} = 5.390 MHz with amplitudes of 1.5 V_{rms} whilst both signal waves are active at f_{s1} = 5.025 MHz and f_{s2} = 5.093 MHz with amplitudes of 2.0 V_{rms}. The degenerate idlers undergo interference when the pump phase is adjusted and the destructive interference at $\pi/2$ or $3\pi/2$ can be exploited to build an XOR gate when both signal waves are active.

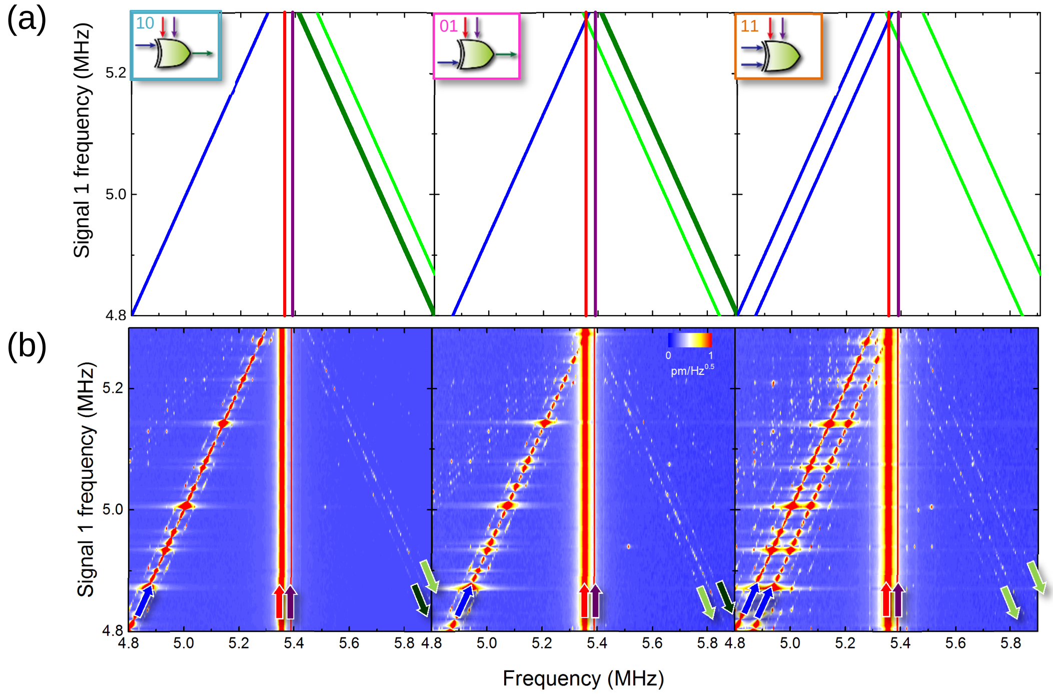

SUPPLEMENTARY FIGURE 7: **XOR gate.** (**a**) The spectral dependence of the idler waves (green and dark green lines), as detailed above, generated from two pump waves with $\pi/2$ phase difference (red and purple lines respectively) at fixed frequencies as function of two signal waves (blue lines) when either is on/off (left panel) or off/on (middle panel) or when both are on (right panel) corresponding to logical inputs 10, 01 and 11 respectively. In the latter configuration, the degenerate idler (dark green line) undergoes destructive interference as detailed in Supplementary Fig. 6 and is eliminated thus yielding an XOR gate. (**b**) The experiments were conducted in region G3 of WG 1 with two 1.5 V_{rms} pump waves fixed at $f_{p1} = 5.356$ MHz and $f_{p2} = 5.390$ MHz i.e. $\Delta = 67.5$ kHz with a $\pi/2$ phase shift (red and purple arrows). Two signals waves (blue arrows) are injected with an amplitude of 2.0 V_{rms} at $f_{s2} = f_{s1} + 67.5$ kHz in the frequency range of 4.8-5.3 MHz encoding to the logical inputs. The degenerate idler waves at $f_{i11} = f_{i22}$ (dark green arrow) observed between 5.45-5.9 MHz is eliminated when both signal waves are activated yielding the XOR gate in the phonon WG. The insets show the signal and pump configurations for the logic gate being performed.
